# First Detection and Molecular Characterization of Novel Variant Infectious Bursal Disease Virus (Genotype A2dB1b) in Egypt

**DOI:** 10.3390/v15122388

**Published:** 2023-12-07

**Authors:** Matteo Legnardi, Francesca Poletto, Shaimaa Talaat, Karim Selim, Mahmoud K. Moawad, Giovanni Franzo, Claudia Maria Tucciarone, Mattia Cecchinato, Hesham Sultan

**Affiliations:** 1 Department of Animal Medicine, Production and Health (MAPS), University of Padova, 35020 Legnaro, Italy; francesca.poletto@unipd.it (F.P.); giovanni.franzo@unipd.it (G.F.); claudiamaria.tucciarone@unipd.it (C.M.T.); mattia.cecchinato@unipd.it (M.C.); 2 Department of Birds and Rabbits Medicine, Faculty of Veterinary Medicine, University of Sadat City, Menoufia 32958, Egypt; shimaa.talaat@vet.usc.edu.eg; 3 Reference Laboratory for Quality Control on Poultry Production, Animal Health Research Institute, Agriculture Research Center, Giza 12618, Egypt; dr.kareemseleem_87@yahoo.com (K.S.); mah.kamel@hotmail.com (M.K.M.)

**Keywords:** infectious bursal disease virus, Gumboro disease, Egypt, China, very virulent, novel variant, molecular epidemiology

## Abstract

Infectious bursal disease (IBD) is an immunosuppressive disease causing significant damage to the poultry industry worldwide. Its etiological agent is infectious bursal disease virus (IBDV), a highly resistant RNA virus whose genetic variability considerably affects disease manifestation, diagnosis and control, primarily pursued by vaccination. In Egypt, very virulent strains (genotype A3B2), responsible for typical IBD signs and lesions and high mortality, have historically prevailed. The present molecular survey, however, suggests that a major epidemiological shift might be occurring in the country. Out of twenty-four samples collected in twelve governorates in 2022–2023, seven tested positive for IBDV. Two of them were A3B2 strains related to other very virulent Egyptian isolates, whereas the remaining five were novel variant IBDVs (A2dB1b), reported for the first time outside of Eastern and Southern Asia. This emerging genotype spawned a large-scale epidemic in China during the 2010s, characterized by subclinical IBD with severe bursal atrophy and immunosuppression. Its spread to Egypt is even more alarming considering that, contrary to circulating IBDVs, the protection conferred by available commercial vaccines appears suboptimal. These findings are therefore crucial for guiding monitoring and control efforts and helping to track the spread of novel variant IBDVs, possibly limiting their impact.

## 1. Introduction

Infectious bursal disease (IBD), also known as Gumboro disease, is an immunosuppressive infectious disease of chickens with severe implications for the global poultry industry. IBD is characterized by high morbidity and mostly occurs in chickens aged 2–6 weeks, when the bursa of Fabricius, its main target organ, reaches its full development. After a short incubation period, the disease typically manifests with non-specific signs, such as depression and dehydration, along with hemorrhagic lesions in the thigh and breast muscles. The bursa appears enlarged at first due to edema and hyperemia but rapidly undergoes atrophy, while lymphocyte depletion is observed at the microscopical level. Alternatively, IBD may follow a subclinical course with lesions limited to the bursa, which may be harder to diagnose whilst still causing immunosuppression [1]. The disease burden may be significant both in case of overt clinical outbreaks and due to the impairment of immune status, which may lead to poor productive performance, vaccine failures, secondary infections, etc. Rigorous control of the disease, primarily pursued by routine vaccination, is therefore of utmost importance [2].

The etiological agent of IBD is known as infectious bursal disease virus (IBDV) and belongs to the species *Avibirnavirus gumboroense*, genus *Avibirnavirus*, family *Birnaviridae*. IBDV features a non-enveloped virion and a double-stranded RNA genome made of two segments, named A and B. Segment A (3.2 kb) codes for a capsid protein (VP2), a scaffold protein (VP3), a protease (VP4) and a non-structural protein with regulatory and anti-apoptotic functions (VP5), whereas segment B (2.9 kb) encodes the RNA-dependent RNA polymerase [3]. Two IBDV serotypes (1 and 2) are known, but only serotype 1 is pathogenic. However, further distinctions are possible, as many different IBDV types have emerged over time, mainly through mutation and reassortment events [4].

As a matter of fact, although IBDV is globally endemic, different countries and regions are affected by a range of viral strains, whose diverse features may have profound consequences in terms of disease manifestation and impact. Historically, the first IBDVs, known as “classical” strains, have been reported in the USA since the late 1950s [5]. Two other IBDV types were then described throughout the 1980s, one grouping highly pathogenic strains (classified as “very virulent”) circulating all over Europe, Africa and Asia, and another comprising the so-called “variant” strains, which were antigenically different from other IBDVs and circulated mainly in North America [6], although they eventually spread to Eastern Asia during the 2010s [7].

In recent years, it has become more and more evident that this traditional tripartite classification, albeit still valuable, is inadequate to fully capture the heterogeneity among IBDV types. Several atypical IBDVs which hardly fit in any of the three major IBDV types have been described in different continents [8,9,10,11,12], and reassortant strains are also being reported with increasing frequency [13,14,15,16], further complicating the evolutionary landscape.

The recent proposal of multiple classification systems relying on phylogeny, either based on a portion of the VP2 [17] or both VP2 and VP1 genes [18,19], has certainly been instrumental for characterizing such strains while retaining the information provided by the traditional classification, offering easily applicable guidelines to perform molecular surveys and generate informative and standardized results. The focus on VP2 and VP1 is motivated by their functional relevance, which makes their genes the most studied genome portions. The VP2 has a well-established role in determining antigenicity, containing the main epitopes that elicit neutralizing antibodies [20], whereas both VP2 and VP1 are known to contribute to pathogenicity determination [21]. Since they are located in different segments, considering both genes also allows to detect reassortment events, which may represent another major source of pathogenic variation [15,22].

The usefulness of this approach is obviously not limited to underinvestigated contexts for which few or no data on circulating IBDVs are available, since it is also helpful to revise the existing evidence and improve monitoring activities even in countries where more information is available. Egypt is certainly an example of the latter case, as the burden posed by IBD to the national poultry production is well-established. Since their first identification in 1989 [23], very virulent strains have consistently posed the greatest threat in the country, as confirmed by several epidemiological studies conducted over the years [24,25,26]. Nonetheless, steady surveillance efforts remain crucial to keep the IBD situation monitored, to assess whether existing control measures are effective and to rapidly identify new epidemiological threats. Consistent with this rationale, the present study reports the results of molecular diagnostic activities performed on samples collected in different Egyptian governorates and contextualizes them within the national and international epidemiological context according to the current classification systems.

## 2. Materials and Methods

### 2.1. Sampling Activities

This study was based on molecular diagnostic activities conducted on samples collected in Egypt for IBD investigation. Samples consisted of bursal imprints on FTA™ cards (GE Healthcare UK Limited, Amersham, UK) and were collected from broiler farms between February 2022 and August 2023 when IBD was suspected based on clinical signs (i.e., anorexia, depression, etc.), lesions (i.e., hemorrhages in the thigh and breast muscles, dehydration, enlarged or atrophic bursa, etc.) and a high mortality rate. Anamnestic information such as farm location, age at sampling, administered IBD vaccines and cumulative mortality up to the sampling date were recorded for all the investigated flocks.

### 2.2. Samples Processing and Nucleic Acid Extraction

Samples were processed by cutting 5 mm^2^ fragments from FTA™ card circles, eluting them into 1.5 mL of 1× PBS and vortexing for 30 s. Nucleic acids were extracted from the eluates by using the High Pure Nucleic Acids kit (Roche™, Basel, Switzerland) following the manufacturer’s instructions. Samples were stored at −80 °C for the entire duration of the molecular analyses and subsequently for archival purposes.

### 2.3. Molecular Investigation

All samples were first subjected to a one-step RT-PCR performed with the primers 743-1 and 743-2 designed by Jackwood and Sommer-Wagner [27] to amplify a portion of the VP2 gene. Additional RT-PCRs were then conducted on positive samples using multiple primer pairs partially derived from those listed by Lachheb et al. [28]. In detail, primers VP5/1+ and VP2/1263- were used to amplify the rest of the VP2 [29], whereas primers 66 and 67 [30], B-Univ-F and B-Univ-R [31], X3 [32] and VP1/1997- [33] and B3-IPP2 and B3′-P2 [34] allowed to cover the entire VP1 gene (Table 1).

The SuperScript™ III One-Step RT-PCR System with Platinum™ *Taq* DNA Polymerase kit (Invitrogen™, Waltham, MA, USA) was used to carry out all molecular assays. Whenever a positive result was evidenced by gel electrophoresis, amplicons were sent to Macrogen Europe Milan Genome Center (Milan, Italy), where Sanger sequencing was performed using the respective primer pair. The resulting chromatograms were visually inspected and appropriately trimmed using 4Peaks (Nucleobytes B.V., Aalsmer, The Netherlands), and then used to generate consensus sequences in ChromasPro (Technelysium Pty Ltd., Helensvale, QLD, Australia).

### 2.4. Phylogenetic Analyses

Sequencing results were used to characterize the detected strains based on the classification system proposed by Wang et al. [19], considering a portion of the hypervariable region (HVR) of the VP2 gene (nt 737–1210) and the B marker located in the VP1 gene (nt 328–756) as defined by Alfonso-Morales et al. [35]. For both genomic segments, along with the reference sequences used by Wang et al. [36], additional strains related to those detected in the present survey, retrieved through dedicated BLAST queries [37], were also considered. After aligning the reference datasets with the MUSCLE method in Mega X [38], phylogenetic trees were inferred using the same software, adopting the Maximum-Likelihood method with 1000 bootstraps and the substitution model having the lowest Bayesian information criterion (BIC) value. The resulting trees were then visualized using the Interactive Tree Of Life online tool [39]. The obtained amino acid sequences were also compared with those of reference isolates when deemed appropriate to identify relevant substitutions.

## 3. Results

A total of 24 samples were collected from broiler farms located in 12 different governorates. The age at sampling was between 18 and 30 days (23.2 days on average). All flocks were reportedly immunized against IBD with a range of vaccination protocols relying on immune complex, vector or live vaccines (sometimes administered twice or after vector vaccines). Seven samples (H792, H793, H798, H800, H801, H805 and H812) tested positive for IBDV (29%). Detailed information on the sampled flocks is provided in Table 2.

**Figure 1:**
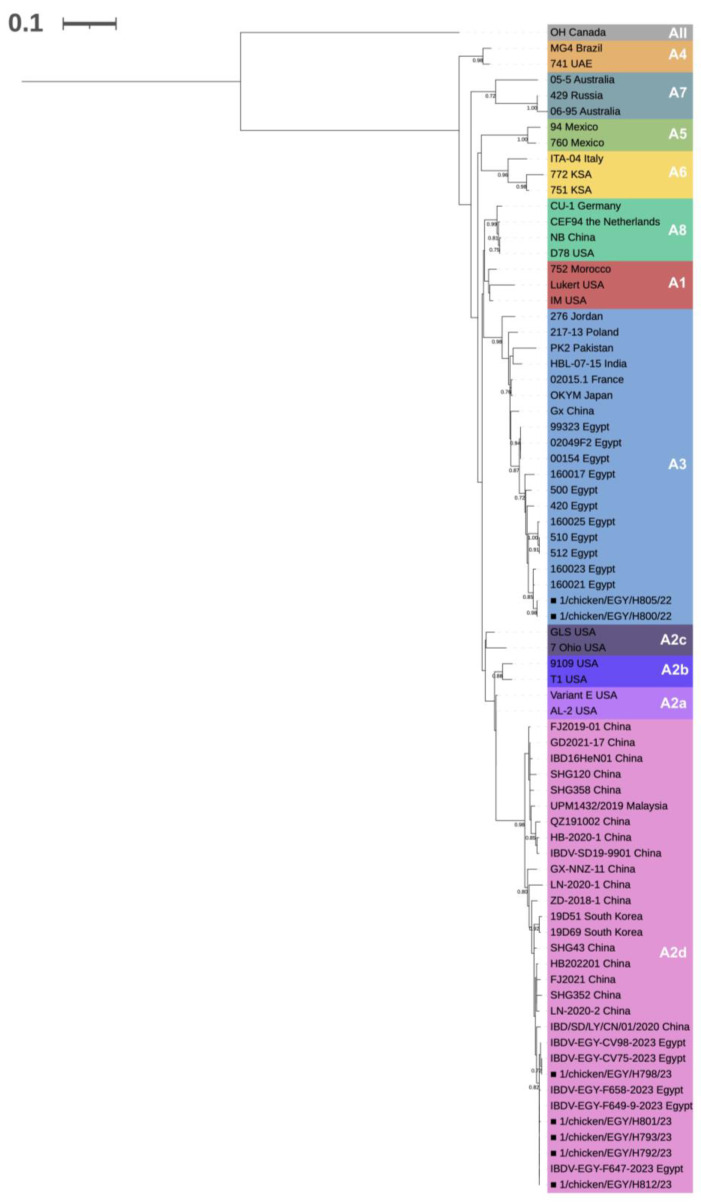
Classification of the detected field strains (marked with solid squares, ■) at VP2 level according to Wang et al. [19]. The evolutionary history was inferred with the Maximum Likelihood Method (1000 bootstraps) applying the K2 + G substitution model [40], based on 74 sequences and considering a 473 nt long portion. Node support values are shown only when higher than 70.

Five of the obtained VP2 sequences showed a reciprocal genetic identity ranging from 99.5 to 100% and belonged to genogroup A2 lineage d (novel variant). The remaining two VP2 sequences were identical to each other and fell within genogroup A3 (very virulent) (Figure 1).

**Figure 2:**
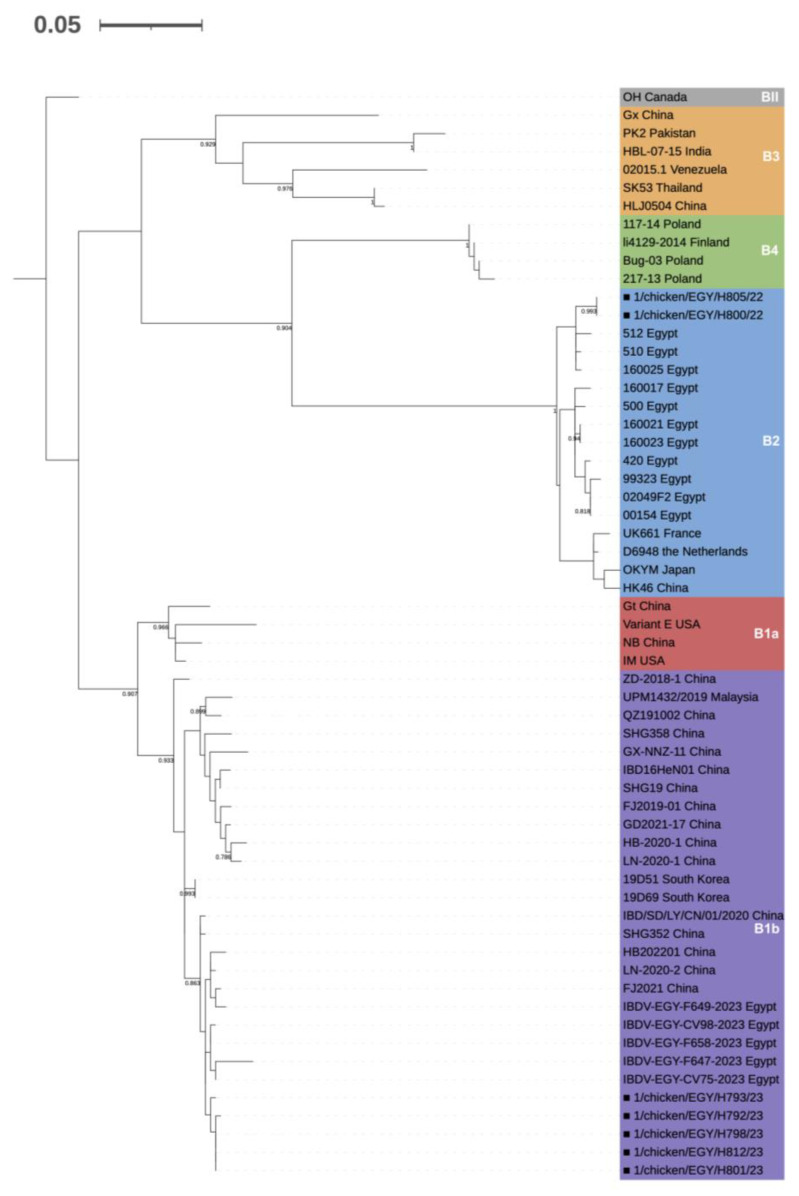
Classification of the detected field strains (marked with solid squares, ■) at VP1 level according to Wang et al. [19]. The evolutionary history was inferred with the Maximum Likelihood Method (1000 bootstraps) applying the K2 + G + I substitution model [40], based on 60 sequences and considering a 428 nt long portion. Node support values are shown only when higher than 70.

The detection of two separate strain clusters was confirmed also at the VP1 level. The five A2d strains had a 99.6–100% reciprocal genetic identity and belonged to VP1 genogroup B1 lineage b (novel variant), whereas the two A3 ones showed a 99.9% identity and were part of genogroup B2 (very virulent) (Figure 2). The two identified genotypes, both having a field origin, were thus A2dB1b and A3B2. In both cases, the most closely related sequences retrieved from GenBank belonged to recent Egyptian isolates. VP1 and VP2 sequences were submitted to GenBank under the accession numbers OR791866-OR791872 and OR79183-OR791879, respectively.

**Figure 3:**
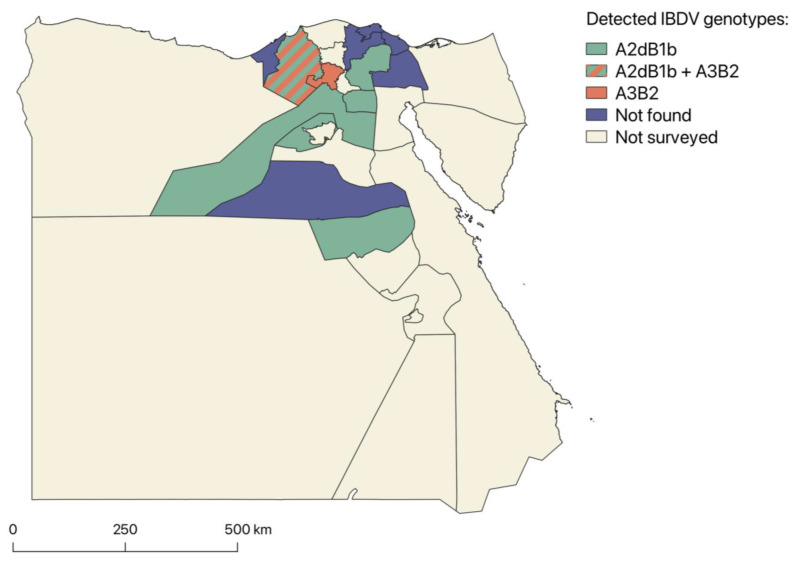
Distribution of IBDV field genotypes at governorate level according to the present molecular survey. The map was prepared using QGIS ver. 3.34 [41] based on a shapefile retrieved from the Dataset of Global Administrative Areas ver. 4.1 (GADM) [42].

Positive samples were collected from farms located in six different governorates, namely Asyut, Beheira, Cairo, Giza, Monufia and Sharqia. No IBDV was detected in the remaining six governorates (Alexandria, Dakahlia, Damietta, Ismailia, Minya and Port Said). The distribution of the two detected genotypes is shown in Figure 3.

The amino acid sequences of the detected A2dB1b strains were compared to those of other novel variant isolates to establish whether any difference was acquired at relevant sites. No consistent amino acid substitutions were unique to Egyptian strains compared to viruses of Asian origin. Nonetheless, multiple changes found in all Egyptian strains, such as A321V at the VP2 level and R511K, S687P and T859I within VP1, had previously only been encountered in a handful of Chinese novel variant IBDVs. Moreover, several substitutions were present only in some of the sequenced strains, both at the VP2 (I15M, S76N, N97K, A277V, G409A) and VP1 level (E393D, T576S, S596F, G630S, Q832R, Q879P) (Supplementary Tables S1 and S2).

## 4. Discussion

Despite the small scale of the study, the present results offer meaningful insights into the circulation of IBDV in Egypt, which, albeit partly in agreement with the established epidemiological scenario, also suggest that a change of great concern may be occurring.

Two of the seven field IBDVs detected were characterized as A3B2 strains, commonly referred to as very virulent strains. The enduring circulation of very virulent IBDVs in Egypt is well documented [23,24,25] and is further corroborated by detections in turkeys [43] and in cattle egret (*Bubulcus ibis*) [44], which suggest that interspecies transmission may play a role in their spread and maintenance. The herein described A3B2 viruses clustered with other Egyptian sequences both at the VP2 and VP1 level, thus confirming the persistence of very virulent strains with consistent features at national level. According to Samy et al. [26], very virulent Egyptian IBDVs can further be divided into antigenically typical and atypical strains based on residue 321 within VP2, with the former group featuring an alanine and the latter a threonine. The two detected strains presented an alanine in that position and can be therefore considered as typical very virulent IBDVs. Although this single mutation was shown to induce drastic changes in reactivity towards neutralizing monoclonal antibodies directed against VP2, it seems to have neither positive nor negative effects on viral fitness, as supported by the long-lasting cocirculation of these two very virulent subtypes in the country [26].

The detection of five novel variant strains, on the other hand, represents an unexpected and alarming finding. These strains have been reported since 2015 in China, where they caused a large-scale epidemic of subclinical IBD [45]. According to phylogenetic analyses, novel variants are related to North American variant IBDVs (genotype A2B1), but also sufficiently divergent to be considered as part of segregated lineage A2d [19]. Similarly, novel variant IBDVs cluster separately from other exponents of genogroup B1, leading to the definition of lineage B1b [35]. Their emergence seems to have been caused by a spread of variant IBDVs from North America to China during the 1990s, followed by a period of latent circulation until A2dB1b broke out in the 2010s [36]. A2dB1b strains were also involved in a reassortment event with an A3B3 IBDV of Chinese origin, originating a novel genotype (A2dB3) showing enhanced pathogenicity [46].

Despite their recent identification, novel variant IBDVs rapidly became one of the dominant IBDV types in Eastern and Southern Asia. Outside of China, they were also found in Malaysia [47], South Korea [48,49] and likely in Japan [50], although the unavailability of Japanese VP1 sequences does not allow to confirm this claim. The spread of novel variant IBDVs to these countries seems to have been favored by strong trade flows of live chickens and poultry products [36], but their entry into Egypt appears more difficult to explain. Interestingly, when the diagnostic activities on which this study is based were originally conducted, all the available A2dB1b sequences with a high genetic identity to the herein described strains were of Chinese origin. However, when the same BLAST query was later repeated, a group of highly homologous Egyptian sequences, which were also collected in 2022 and 2023 based on their metadata, were also retrieved. This information clearly substantiates the present results, suggesting that novel variant strains might be affirming themselves as a significant epidemiological threat in Egypt despite an apparently recent entry in the country.

The comparison of amino acid sequences did not highlight any unique substitution in the Egyptian strains. Nonetheless, some of the observed changes were only present in a minority of Asian novel variant IBDVs. Like in the case of Egyptian A3B2 strains, the most notable mutation involved residue 321 of the VP2, located in the P^HI^ loop and supposedly involved in antigenicity determination [20,26]. The A321V substitution observed in most Egyptian novel variant IBDVs was found only in Chinese strain IBD/SD/LY/CN/01/2020, whereas other novel variant viruses showed an A321T change, corroborating previous reports that this site may be prone to mutations [17]. At the VP1 level, Egyptian novel variant IBDVs showed three consistent substitutions compared to most A2dB1b strains, namely R511K, S687P and T859I. Among these, residue 687 has been proposed to play a role in the increased pathogenicity of very virulent IBDVs, which feature a proline, compared to less virulent strains, mostly featuring a serine [51]. In future studies, it will be important to monitor the evolution of Egyptian novel variant IBDVs to determine if these changes will become a permanent feature and if others will emerge as a possible consequence to the adaptation to a new epidemiological context.

Currently, novel variant strains do not seem to be circulating in countries neighboring Egypt. A recent epidemiological survey conducted in the Near and Middle East highlighted the circulation of A3B1, A3B2, A4B1 and A6B1 IBDVs [52], whereas the most recent studies conducted in Northeast Africa suggest that very virulent IBDVs still represent the main threat in the area [53,54]. Nonetheless, further monitoring activities, to be conducted not only in Egypt but also in countries where A2dB1b strains may be circulating undetected, are required to shed light on their diffusion and to track their potential spread to new territories.

Both A3B2 and A2dB1b were found in multiple governorates in the northern and central part of Egypt. The limited number of samples collected in different governorates meant that usually only one of the two genotypes was detected in each of them, with Beheira being the only exception. However, considering that very virulent IBDVs are widespread in the country and that the investigated governorates were in proximity to each other, it might be assumed that very virulent and variant strains are cocirculating in the same areas. It is also worth noting that field IBDVs have already been detected in all the governorates from which no positive samples were retrieved [24,26,55,56,57,58], suggesting that this finding was due to the small sample size rather than the actual absence of field strains in these settings.

Aside from characterizing field strains from a molecular perspective, it is essential to understand their pathogenic features and ensure that the currently enacted control measures are effective. A wealth of data has been produced in the Egyptian context on the pathogenicity of locally circulating very virulent IBDVs, which consistently cause typical IBD signs and lesions with high mortality rates [59,60,61], and on the protection induced by different live [61], vector [62,63] and immune complex [64] vaccines, which appears adequate. Novel variant IBDVs, on the other hand, are associated with subclinical infections with severe bursal atrophy and lymphocyte depletion [16,46,65,66]. Infections sustained by these strains may therefore be easily overlooked, favoring their spread and circulation. Another factor that likely played a role in their evolutionary success is their divergent antigenic features, which may thwart control measures. As a matter of fact, the effectiveness of currently marketed vaccines against them has been put into question [67,68]. This prompted the development of multiple vaccine candidates based on different technologies, including reassortment [67] and virus-like particle vaccines [69,70,71], which yielded promising results in terms of efficacy and safety but are not yet commercially available.

In partial contrast with the literature, the anamnestic information retrieved during sampling activities suggests that both very virulent and novel variant IBDVs were responsible for severe mortality. This finding might be explained by the immunosuppressive potential of A2dB1b strains, which may have favored secondary infections and reduced the efficacy of vaccination against relevant diseases affecting the Egyptian poultry sector, including avian influenza and Newcastle disease [72]. Nonetheless, it should be noted that equally high or even higher mortality rates were also encountered in IBDV-negative flocks, and that no other possible cause (neither primary nor secondary) was investigated, limiting such conclusions. Since the present research was not originally designed with this aim, additional studies, which should include viral isolation and standardized experimental infections, are therefore required to properly evaluate the pathogenic features of Egyptian novel variant strains.

The complete absence of IBDV vaccine detections represents another noteworthy finding. Detecting the administered vaccine strains is commonly considered a useful proxy for vaccine take and coverage, particularly in the case of vaccines relying on bursal colonization (i.e., live and immune complex vaccines) and to a lesser extent for vector vaccines expressing VP2 inserts, which can still be found in the bursa, although it is not their primary replication site [73]. Even if the early sampling age likely hampered vaccine detection in some cases, especially when live vaccines were used, the absence of vaccine-positive flocks suggests that the conferred protection might have been subpar, and that administration errors at hatchery or farm level cannot be excluded. On this note, many of the sampled farms were anecdotally reported to have a multi-age organization and to suffer from managerial and hygiene deficiencies, thus complicating vaccine administration and increasing the risk of exposure to field IBDVs. Regardless of the circulating field genotypes and used vaccine types, the optimization of vaccination quality and its continuous assessment should represent a priority not only to protect the immunized flock against clinical signs, but also to reduce the circulation and persistence of field viruses in the long term, eventually favoring the entire epidemiological scenario rather than just the single immunized flock.

## 5. Conclusions

The present study provides a crucial update on the IBDV epidemiological situation in Egypt, capturing the entry of novel variant strains in the country in a timely manner. Compared to the historically circulating very virulent IBDVs, which were still detected, such viruses, reported for the first time outside of Asia, pose an entirely different challenge both in terms of clinical manifestation, as they are mostly subclinical and thus easily overlooked despite still causing relevant losses, and required control measures, as the protection conferred by the currently marketed vaccines is likely limited by their antigenic divergence. Albeit relevant, the identification and molecular characterization of genotype A2dB1b should be intended as just the first step of a larger-scoped investigation. Its spread to Egypt could lead to its establishment as a substantial epidemiological threat in the country and neighboring regions, requiring appropriate studies to track its propagation and evolution, establish its pathogenicity and ultimately assess its impact in a different epidemiological context from the one where it originated.

## Figures and Tables

**Table 1:** List of primer pairs used for the amplification and sequencing of the VP2 and VP1 genes.

Genome Segment	Primer	Sequence (5′-3′)	Amplicon Length	Designed by
VP5 and VP2 (1–1263)	VP5/1+	GGATACGATCGGTCTGAC	1263 bp	Hernández et al. [29]
	VP2/1263-	TCAGGATTTGGGATCAGC		
VP2 (736–1478)	743-1	GCCCAGAGTCTACACCAT	743 bp	Jackwood and Sommer-Wagner [27]
	743-2	CCCGGATTATGTCTTTGA		
VP1 (1–695)	66	GGATACGATGGGTCTGAC	695 bp	Ruud et al. [30]
	67	ATCCTTGACGGCACCCTT		
VP1 (319–1369)	B-Univ-F	AATGAGGAGTATGAGACCGA	1051 bp	Islam et al. [31]
	B-Univ-R	CCTTCTCTAGGTCAATTGAGTACC		
VP1 (756–1997)	X3	CGGTGAGGATGACAAGCCC	1241 bp	He et al. [32]
	VP1/1997-	GAACCCCTTTGCCTCCAAG		Tiwari et al. [33]
VP1 (1839–2827)	B3-IPP2	ATACAGCAAAGATCTCGGG	988 bp	Mundt and Vakharia [34]
	B3′-P2	CGATCTGCTGCAGGGGGCCCCCGCAGGCGAAGG		

**Table 2:** Anamnestic details recorded for each of the sampled flocks.

Sample ID	Collection Date	Farm Location	Age at Sampling	Vaccination Protocol	Mortality *	IBDV Result
H792	February 2023	Cairo	19 d	1 d: vector vaccine	11.2	A2dB1b
H793	March 2023	Giza	21 d	1 d: vector vaccine; 14 d: live vaccine	9.7	A2dB1b
H794	April 2023	Alexandria	23 d	1 d: vector vaccine	13	Negative
H795	August 2022	Damietta	21 d	1d: vector vaccine; 12 d: live vaccine	13	Negative
H796	June 2022	Beheira	25 d	1 d: vector vaccine; 14 d: live vaccine	10.5	Negative
H797	January 2023	Cairo	28 d	14 d: live vaccine; 18 d: live vaccine	12.7	Negative
H798	July 2023	Sharqia	22 d	1 d: vector vaccine; 14 d: live vaccine	9.6	A2dB1b
H799	December 2022	Giza	30 d	12 d: live vaccine; 18 d: live vaccine	22.7	Negative
H800	April 2022	Beheira	24 d	1 d: vector vaccine; 12 d: live vaccine	17	A3B2
H801	August 2023	Asyut	18 d	12 d: live vaccine	9.8	A2dB1b
H802	February 2023	Dakahlia	22 d	1 d: vector vaccine	11.6	Negative
H803	March 2023	Sharqia	25 d	1 d: immune complex vaccine	8	Negative
H804	March 2023	Sharqia	22 d	1 d: immune complex vaccine	12.4	Negative
H805	February 2022	Monufia	26 d	12 d: live vaccine; 18 d: live vaccine	13.8	A3B2
H806	February 2022	Dakahlia	26 d	1 d: vector vaccine	13.9	Negative
H807	September 2023	Minya	21 d	1 d: vector vaccine; 14 d: live vaccine	9	Negative
H809	July 2022	Alexandria	20 d	12 d: live vaccine; 20 d: live vaccine	8	Negative
H810	June 2023	Giza	27 d	1 d: immune complex vaccine	16	Negative
H811	April 2022	Giza	24 d	1 d: immune complex vaccine	11.5	Negative
H812	August 2023	Beheira	19 d	1 d: vector vaccine; 12 d: live vaccine	8.7	A2dB1b
H813	February 2022	Ismailia	24 d	1 d: vector vaccine; 12 d: live vaccine	14	Negative
H814	April 2023	Ismailia	20 d	1 d: vector vaccine; 12 d: live vaccine	10.7	Negative
H815	June 2022	Port Said	23 d	1 d: vector vaccine	11	Negative

* Cumulative mortality observed from the start of the productive cycle to the sampling date.

## Data Availability

Data are contained within the article and Supplementary Materials.

## Supplementary Materials

The following supporting information can be downloaded at: https://www.mdpi.com/article/10.3390/v15122388/s1, Table S1: Comparison of VP2 amino acid sequences of A2dB1b strains; Table S2: Comparison of VP1 amino acid sequences of A2dB1b strains.

## References

[1] Eterradossi N., Saif Y.M., Swayne D.E., Boulianne M., Logue C.M., McDougald L.R., Nair V., Suarez D.L. (2020). Infectious bursal disease. Diseases of Poultry.

[2] Alkie T.N., Rautenschlein S. (2016). Infectious bursal disease virus in poultry: Current status and future prospects. Vet. Med..

[3] Maraver A., Ona A., Abaitua F., Gonzalez D., Clemente R., Ruiz-Diaz J.A., Caston J.R., Pazos F., Rodriguez J.F. (2003). The oligomerization domain of VP3, the scaffolding protein of infectious bursal disease virus, plays a critical role in capsid assembly. J. Virol..

[4] Pikuła A., Lisowska A., Jasik A., Perez L.J. (2021). The Novel Genetic Background of Infectious Bursal Disease Virus Strains Emerging from the Action of Positive Selection. Viruses.

[5] Cosgrove A.S. (1962). An apparently new disease of chickens: Avian nephrosis. Avian Dis..

[6] Lasher H., Davis V. (1997). History of infectious bursal disease in the U.S.A.: The first two decades. Avian Dis..

[7] Zhang W., Wang X., Gao Y., Qi X. (2022). The Over-40-years-epidemic of infectious bursal disease virus in China. Viruses.

[8] Sapats S.I., Ignjatovic J. (2000). Antigenic and sequence heterogeneity of infectious bursal disease virus strains isolated in Australia. Arch. Virol..

[9] Jackwood D.J. (2012). Molecular epidemiologic evidence of homologous recombination in infectious bursal disease viruses. Avian Dis..

[10] Hernández M., Tomás G., Marandino A., Iraola G., Maya L., Mattion N., Hernández D., Villegas P., Banda A., Panzera Y. (2015). Genetic characterization of South American infectious bursal disease virus reveals the existence of a distinct worldwide-spread genetic lineage. Avian Pathol..

[11] Lupini C., Giovanardi D., Pesente P., Bonci M., Felice V., Rossi G., Morandini E., Cecchinato M., Catelli E. (2016). A molecular epidemiology study based on VP2 gene sequences reveals that a new genotype of infectious bursal disease virus is dominantly prevalent in Italy. Avian Pathol..

[12] Legnardi M., Franzo G., Tucciarone C.M., Koutoulis K., Duarte I., Silva M., Le Tallec B., Cecchinato M. (2022). Detection and molecular characterization of a new genotype of infectious bursal disease virus in Portugal. Avian Pathol..

[13] Abed M., Soubies S., Courtillon C., Briand F.X., Allée C., Amelot M., De Boisseson C., Lucas P., Blanchard Y., Belahouel A. (2018). Infectious bursal disease virus in Algeria: Detection of highly pathogenic reassortant viruses. Infect. Genet. Evol..

[14] Pikuła A., Lisowska A., Jasik A., Śmietanka K. (2018). Identification and assessment of virulence of a natural reassortant of infectious bursal disease virus. Vet. Res..

[15] Mató T., Tatár-Kis T., Felföldi B., Jansson D.S., Homonnay Z., Bányai K., Palya V. (2020). Occurrence and spread of a reassortant very virulent genotype of infectious bursal disease virus with altered VP2 amino acid profile and pathogenicity in some European countries. Vet. Microbiol..

[16] Wang Y., Jiang N., Fan L., Niu X., Zhang W., Huang M., Gao L., Li K., Gao Y., Liu C. (2021). Identification and Pathogenicity Evaluation of a Novel Reassortant Infectious Bursal Disease Virus (Genotype A2dB3). Viruses.

[17] Michel L.O., Jackwood D.J. (2017). Classification of infectious bursal disease virus into genogroups. Arch. Virol..

[18] Islam M.R., Nooruzzaman M., Rahman T., Mumu T.T., Rahman M.M., Chowdhury E.H., Eterradossi N., Müller H. (2021). A unified genotypic classification of infectious bursal disease virus based on both genome segments. Avian Pathol..

[19] Wang Y.L., Fan L.J., Jiang N., Li G.A.O., Kai L.I., Gao Y.L., Liu C.J., Cui H.Y., Pan Q., Zhang Y.P. (2021). An improved scheme for infectious bursal disease virus genotype classification based on both genome-segments A and B. J. Integr. Agric..

[20] Letzel T., Coulibaly F., Rey F.A., Delmas B., Jagt E., Van Loon A.A., Mundt E. (2007). Molecular and structural bases for the antigenicity of VP2 of infectious bursal disease virus. J. Virol..

[21] Escaffre O., Le Nouën C., Amelot M., Ambroggio X., Ogden K.M., Guionie O., Toquin D., Müller H., Islam M.R., Eterradossi N. (2013). Both genome segments contribute to the pathogenicity of very virulent infectious bursal disease virus. J. Virol..

[22] He X., Chen G., Yang L., Xuan J., Long H., Wei P. (2016). Role of naturally occurring genome segment reassortment in the pathogenicity of IBDV field isolates in Three-Yellow chickens. Avian Pathol..

[23] El-Batrawy A. Studies on Severe Outbreaks of Infectious Bursal Disease. Proceedings of the 2nd Scientific Conference of the Egyptian Veterinary Poultry Association.

[24] Mawgod S.A., Arafa A.S., Hussein H.A. (2014). Molecular genotyping of the infectious bursal disease virus (IBDV) isolated from broiler flocks in Egypt. Int. J. Vet. Sci. Med..

[25] Shehata A.A., Sultan H., Halami M.Y., Talaat S., Vahlenkamp T.W. (2017). Molecular characterization of very virulent infectious bursal disease virus strains circulating in Egypt from 2003 to 2014. Arch. Virol..

[26] Samy A., Courtillon C., Briand F.X., Khalifa M., Selim A., Hegazy A., Eterradossi N., Soubies S.M. (2020). Continuous circulation of an antigenically modified very virulent infectious bursal disease virus for fifteen years in Egypt. Infect. Genet. Evol..

[27] Jackwood D.J., Sommer-Wagner S.E. (2005). Molecular Epidemiology of Infectious Bursal Disease Viruses: Distribution and Genetic Analysis of Newly Emerging Viruses in the United States. Avian Dis..

[28] Lachheb J., Jbenyeni A., Nsiri J., Larbi I., Ammouna F., Ghram A. (2021). Full-length genome sequencing of a very virulent infectious bursal disease virus isolated in Tunisia. Poult. Sci..

[29] Hernández M., Villegas P., Hernández D., Banda A., Maya L., Romero V., Tomás G., Pérez R. (2010). Sequence variability and evolution of the terminal overlapping VP5 gene of the infectious bursal disease virus. Virus Genes.

[30] Rudd M.F., Heine H.G., Sapats S.I., Parede L., Ignjatovic J. (2002). Characterisation of an Indonesian very virulent strain of infectious bursal disease virus. Arch. Virol..

[31] Islam M.R., Rahman S., Noor M., Chowdhury E.H., Müller H. (2011). Differentiation of infectious bursal disease virus (IBDV) genome segment B of very virulent and classical lineage by RT-PCR amplification and restriction enzyme analysis. Arch. Virol..

[32] He X., Xiong Z., Yang L., Guan D., Yang X., Wei P. (2014). Molecular epidemiology studies on partial sequences of both genome segments reveal that reassortant infectious bursal disease viruses were dominantly prevalent in southern China during 2000–2012. Arch. Virol..

[33] Tiwari A.K., Kataria R.S., Prasad N., Gupta R. (2003). Differentiation of infectious bursal disease viruses by restriction enzyme analysis of RT-PCR amplified VP1 gene sequence. Comp. Immunol. Microbiol. Infect. Dis..

[34] Mundt E., Vakharia V.N. (1996). Synthetic transcripts of double-stranded Birnavirus genome are infectious. Proc. Natl. Acad. Sci. USA.

[35] Alfonso-Morales A., Rios L., Martínez-Pérez O., Dolz R., Valle R., Perera C.L., Bertran K., Frias M.T., Ganges L., Diaz de Arce H. (2015). Evaluation of a phylogenetic marker based on genomic segment B of infectious bursal disease virus: Facilitating a feasible incorporation of this segment to the molecular epidemiology studies for this viral agent. PLoS ONE.

[36] Wang W., He X., Zhang Y., Qiao Y., Shi J., Chen R., Chen J., Xiang Y., Wang Z., Chen G. (2022). Analysis of the global origin, evolution and transmission dynamics of the emerging novel variant IBDV (A2dB1b): The accumulation of critical aa-residue mutations and commercial trade contributes to the emergence and transmission of novel variants. Transbound. Emerg. Dis..

[37] Altschul S.F., Gish W., Miller W., Myers E.W., Lipman D.J. (1990). Basic local alignment search tool. J. Mol. Biol..

[38] Kumar S., Stecher G., Li M., Knyaz C., Tamura K. (2018). MEGA X: Molecular evolutionary genetics analysis across computing platforms. Mol. Biol. Evol..

[39] Letunic I., Bork P. (2021). Interactive Tree Of Life (iTOL) v5: An online tool for phylogenetic tree display and annotation. Nucleic Acids Res..

[40] Kimura M. (1980). A simple method for estimating evolutionary rates of base substitutions through comparative studies of nucleotide sequences. J. Mol. Evol..

[41] QGIS. https://www.qgis.org/.

[42] Database of Global Administrative Areas (GADM). https://gadm.org/.

[43] Mosad S.M., Eladl A.H., El-Tholoth M., Ali H.S., Hamed M.F. (2020). Molecular characterization and pathogenicity of very virulent infectious bursal disease virus isolated from naturally infected Turkey poults in Egypt. Trop. Anim. Health Prod..

[44] El Naggar R.F., Rohaim M.A., Munir M. (2020). Potential reverse spillover of infectious bursal disease virus at the interface of commercial poultry and wild birds. Virus Genes.

[45] Fan L., Wu T., Hussain A., Gao Y., Zeng X., Wang Y., Gao L., Li K., Wang Y., Liu C. (2019). Novel variant strains of infectious bursal disease virus isolated in China. Vet. Microbiol..

[46] Wang W., Huang Y., Zhang Y., Qiao Y., Deng Q., Chen R., Chen J., Huang T., Wei T., Mo M. (2022). The emerging naturally reassortant strain of IBDV (genotype A2dB3) having segment A from Chinese novel variant strain and segment B from HLJ 0504-like very virulent strain showed enhanced pathogenicity to three-yellow chickens. Transbound. Emerg. Dis..

[47] Aliyu H.B., Hair-Bejo M., Omar A.R., Ideris A. (2021). Genetic diversity of recent infectious bursal disease viruses isolated from vaccinated poultry flocks in Malaysia. Front. Vet. Sci..

[48] Thai T.N., Jang I., Kim H.A., Kim H.S., Kwon Y.K., Kim H.R. (2021). Characterization of antigenic variant infectious bursal disease virus strains identified in South Korea. Avian Pathol..

[49] Thai T.N., Yoo D.S., Jang I., Kwon Y.K., Kim H.R. (2022). Dynamics of the Emerging Genogroup of Infectious Bursal Disease Virus Infection in Broiler Farms in South Korea: A Nationwide Study. Viruses.

[50] Myint O., Suwanruengsri M., Araki K., Izzati U.Z., Pornthummawat A., Nueangphuet P., Fuke N., Hirai T., Jackwood D.J., Yamaguchi R. (2021). Bursa atrophy at 28 days old caused by variant infectious bursal disease virus has a negative economic impact on broiler farms in Japan. Avian Pathol..

[51] Ren X., Xue C., Zhang Y., Chen F., Cao Y. (2009). Genomic analysis of one Chinese strain YS07 of infectious bursal disease virus reveals unique genetic diversity. Virus Genes.

[52] Legnardi M., Poletto F., Alam S., Cherfane A., Le-Tallec B., Franzo G., Tucciarone C.M., Lupini C., Pasotto D., Cecchinato M. (2023). Molecular epidemiology of infectious bursal disease virus in the Near East and Persian Gulf regions. Avian Pathol..

[53] Shegu D., Sori T., Tesfaye A., Belay A., Mohammed H., Degefa T., Getachew B., Abayneh T., Gelaye E. (2020). Sequence-based comparison of field and vaccine strains of infectious bursal disease virus in Ethiopia reveals an amino acid mismatch in the immunodominant VP2 protein. Arch. Virol..

[54] Omer M.G., Khalafalla A.I. (2022). Epidemiology and laboratory diagnosis of very virulent infectious bursal disease virus in vaccinated chickens in Khartoum, Sudan. Open Vet. J..

[55] Ramzy N., Abdel-fattah S. (2015). Prevalence and molecular characterization of Gumboro virus in chicken farms in Ismailia. Assiut Vet. Med. J..

[56] Omar S.E., El Sayed W.A.E.M., Abdelhalim A., Yehia N. (2021). Genetic evolution of infectious bursal disease virus isolated from chicken poultry flocks in Egypt. J. World Poult. Res..

[57] Awad N., Morsi H., Eid A.A., Al-baqir A. (2023). Epidemiological Occurrence of the Infectious Bursal Disease Virus in Chickens’ Flocks that Had Received Various Vaccination Regimens. Zagazig Vet. J..

[58] Shahat D.H. (2023). Detection and isolation of a recent infectious bursal disease virus from chicken farms in Egypt during 2021. Benha Vet. Med. J..

[59] Suliman R.A., Ahmed B.M., El-Safty M.M., Hussien H.A. (2017). Very virulent IBDV strain Egypt/iBDV/Behera/2011: Macroscopic and microscopic lesions accompanied with induced mortalities in SPF chicks. J. Virol. Sci..

[60] Gaber H.A.H., El-Dougdoug K.A., El-Masry S.S. (2021). Isolation and Pathotyping of Infectious Bursal Disease Virus (IBDV) from Field Outbreaks among Chickens in Egypt. J. Anim. Poult. Prod..

[61] Hassan M.K., Afify M., Aly M.M. (2022). Susceptibility of vaccinated and unvaccinated Egyptian chickens to very virulent infectious bursal disease virus. Avian Pathol..

[62] Sultan H., Hussein H.A., Abd El-Razik A.G., El-Balall S., Talaat S.M., Shehata A.A. (2012). Efficacy of HVT-IBDV vector vaccine against recent Egyptian vvIBDV in commercial broiler chickens. Int. J. Poult. Sci..

[63] Rade N., Sultan H., El-Razik A. (2020). Efficacy of The Turkey Herpes virus-IBDV Vector Vaccine Against Recent Egyptian Very Virulent IBDV in Commercial Layers. J. Curr. Vet. Res..

[64] Eliwa M.G.E.D., Talaat S., Tantawy L., El-Razik A., Sultan H. (2022). Protective efficacy of IBDV winterfield H-2512 and SYZA-26 immune-complex vaccines against recent Egyptian very virulent IBDV in commercial broiler chickens. J. Curr. Vet. Res..

[65] Xu A., Pei Y., Zhang K., Xue J., Ruan S., Zhang G. (2020). Phylogenetic analyses and pathogenicity of a variant infectious bursal disease virus strain isolated in China. Virus Res..

[66] Lian J., Wang Z., Xu Z., Pang Y., Leng M., Tang S., Zhang X., Qin J., Chen F., Lin W. (2022). Pathogenicity and molecular characterization of infectious bursal disease virus in China. Poult. Sci..

[67] Hou B., Wang C.Y., Luo Z.B., Shao G.Q. (2022). Commercial vaccines used in China do not protect against a novel infectious bursal disease virus variant isolated in Fujian. Vet. Rec..

[68] Fan L., Wu T., Wang Y., Hussain A., Jiang N., Gao L., Li K., Gao Y., Liu C., Cui H. (2020). Novel variants of infectious bursal disease virus can severely damage the bursa of fabricius of immunized chickens. Vet. Microbiol..

[69] Fan L., Wang Y., Jiang N., Gao L., Li K., Gao Y., Cui H., Pan Q., Liu C., Zhang Y. (2020). A reassortment vaccine candidate of the novel variant infectious bursal disease virus. Vet. Microbiol..

[70] Li G., Kuang H., Guo H., Cai L., Chu D., Wang X., Hu J., Rong J. (2020). Development of a recombinant VP2 vaccine for the prevention of novel variant strains of infectious bursal disease virus. Avian Pathol..

[71] Wang Y., Jiang N., Fan L., Gao L., Li K., Gao Y., Niu X., Zhang W., Cui H., Liu A. (2021). Development of a Viral-Like Particle Candidate Vaccine Against Novel Variant Infectious Bursal Disease Virus. Vaccines.

[72] El-Aried T.A., Mansour S.M., El Bakrey R.M., Ismail A.E.N., Eid A.A. (2019). Infectious bursal disease virus: Molecular epidemiologic perspectives and impact on vaccine efficacy against avian influenza and Newcastle disease viruses. Avian Dis..

[73] Ramon G., Legnardi M., Cecchinato M., Cazaban C., Tucciarone C.M., Fiorentini L., Gambi L., Mato T., Berto G., Koutoulis K. (2022). Efficacy of live attenuated, vector and immune complex infectious bursal disease virus (IBDV) vaccines in preventing field strain bursa colonization: A European multicentric study. Front. Vet. Sci..

