# Supplementary material for: First Detection and Molecular Characterization of Novel Variant Infectious Bursal Disease Virus (Genotype A2dB1b) in Egypt
Source: Viruses. 2023 Dec 7;15(12):2388. doi: 10.3390/v15122388 (PMC10747051; doi:10.3390/v15122388)
Supplement: Supplementary file 1 [file viruses-15-02388-s001.zip › viruses-2742753-supplementary.pdf]

Supplementary Table S1. Comparison of the VP2 amino acid sequences of the detected A2dB1b strains (marked with solid squares, ■) with relevant novel variant IBDVs. All sites featuring at least one substitution (highlighted in orange) are shown.

		VP2																		
Strain	Country	15	73	76	77	79	97	146	170	187	220	251	277	299	321	336	343	359	405	409
■ 1/chicken/EGY/H792/23	Egypt	I	T	S	D	N	K	S	P	I	Y	S	A	S	V	T	P	K	R	G
■ 1/chicken/EGY/H793/23	Egypt	I	T	S	D	N	K	S	P	I	Y	S	A	S	V	T	P	K	R	G
■ 1/chicken/EGY/H798/23	Egypt	I	T	S	D	N	N	S	P	I	Y	S	A	S	V	T	P	K	R	A
■1/chicken/EGY/H801/2 3	Egypt	I	T	N	D	N	N	S	P	I	Y	S	V	S	V	T	P	K	R	G
■ 1/chicken/EGY/H812/23	Egypt	M	T	N	D	N	N	S	P	I	Y	S	A	S	V	T	P	K	R	G
IBDV-EGY-CV75-2023	Egypt	I	T	S	D	N	N	S	P	I	Y	S	A	S	V	T	P	K	R	G
IBDV-CV98-2023	Egypt	I	T	S	D	N	N	S	P	I	Y	S	A	S	V	T	P	K	R	G
IBDV-EGY-F427-2023	Egypt	I	T	S	D	N	N	S	P	I	Y	S	A	S	A	T	P	K	R	G
IBDV-EGY-F647-2023	Egypt	I	T	S	D	N	N	S	P	I	Y	S	A	S	V	T	P	K	R	G
IBDV-EGY-F649-9-2023	Egypt	I	T	S	D	N	N	S	A	I	Y	S	A	S	A	T	P	K	R	G
IBDV-EGY-F658-2023	Egypt	I	T	S	D	N	N	S	P	I	Y	S	A	S	V	T	P	K	R	G
IBD/SD/LY/CN/01/2020	China	I	T	S	D	N	N	S	P	I	Y	S	A	S	V	T	P	K	R	G
HB202201	China	I	T	S	D	N	N	S	P	I	Y	S	A	S	A	T	P	K	R	G
SHG352	China	I	T	S	D	N	N	S	P	I	Y	S	A	S	A	T	P	K	R	G
IBDV-LY21	China	I	T	S	D	N	N	F	P	I	Y	S	A	S	A	A	S	K	R	G
FJ2021	China	I	T	S	D	N	N	S	P	I	Y	S	A	S	A	T	P	K	R	G
ZD-2018-1	China	I	T	S	D	N	N	S	P	I	Y	S	A	S	A	T	P	K	R	G
LN-2020-2	China	I	T	S	N	N	N	S	P	I	Y	S	A	S	A	T	P	K	R	G
19D69	South Korea	I	T	S	D	S	N	S	P	I	Y	S	A	S	A	T	P	T	R	G
19D51	South Korea	I	T	S	N	S	N	S	P	I	Y	S	A	S	A	T	P	T	R	G
IBD16Hen01	China	I	I	S	D	S	N	S	P	V	Y	S	A	S	A	T	P	T	R	G
SHG19	China	I	I	S	D	S	N	S	P	V	Y	S	A	S	A	T	P	T	R	G
FJ2019	China	I	I	S	D	S	N	S	P	V	Y	S	A	S	A	T	P	K	R	G
SHG358	China	I	I	S	D	S	N	S	P	V	Y	S	A	S	A	T	P	T	R	G
GD2021-17	China	I	I	S	D	S	N	S	P	V	Y	S	A	S	A	T	P	T	R	G
Hb06v	China	I	I	S	D	S	N	S	P	V	Y	S	A	S	A	T	P	T	R	G
GD-1	China	I	I	S	D	S	N	S	P	V	Y	S	A	G	A	T	P	T	R	G
LN-2020-1	China	I	I	S	D	S	N	S	P	V	F	S	A	S	A	T	P	K	R	G
FJ-18	China	I	I	S	D	S	N	S	P	V	Y	S	A	S	A	T	P	K	R	G
QZ191002	China	I	I	S	D	S	N	S	P	V	Y	N	A	S	T	T	P	T	R	G
UPM1432/2019	Malaysia	I	I	S	D	S	N	S	P	V	Y	S	A	S	A	T	P	T	R	G
HB-2020-1	China	I	I	S	D	S	N	S	P	V	Y	S	A	S	T	T	P	T	R	G
IBDV-JS19-14701	China	I	I	S	D	S	N	S	P	V	Y	S	A	S	A	T	P	T	R	G
FJ-1812	China	I	I	S	D	S	N	S	P	V	Y	S	A	S	A	T	P	K	G	G

Supplementary Table S2. Comparison of the VP1 amino acid sequences of the detected A2dB1b strains (marked with solid squares, ■) with relevant novel variant IBDVs. All sites featuring at least one substitution (highlighted in orange) are shown.

		VP1																																								
**Strain**	**Country**	**21**	**54**	**73**	**89**	**90**	**94**	**104**	**107**	**108**	**141**	**146**	**148**	**150**	**239**	**308**	**345**	**393**	**411**	**444**	**456**	**511**	**515**	**532**	**576**	**579**	**595**	**596**	**630**	**687**	**688**	**700**	**718**	**748**	**751**	**766**	**775**	**851**	**859**	**872**	**877**	**879**
■ 1/chicken/EGY/H792/23	Egypt	K	L	Y	E	G	K	Q	F	P	I	E	L	D	A	S	H	E	T	N	F	K	D	R	T	K	C	S	G	P	E	K	N	V	K	D	T	R	I	Q	S	Q
■ 1/chicken/EGY/H793/23	Egypt	K	L	Y	E	G	K	Q	F	P	I	E	L	D	A	S	H	E	T	N	F	K	D	R	T	K	C	S	G	P	E	K	N	V	K	D	T	R	I	Q	S	Q
■ 1/chicken/EGY/H798/23	Egypt	K	L	Y	E	G	K	Q	F	P	I	E	L	D	A	S	H	D	T	N	F	K	D	R	T	K	C	F	S	P	E	K	N	V	K	D	T	R	I	Q	S	Q
■ 1/chicken/EGY/H801/23	Egypt	K	L	Y	E	G	K	Q	F	P	I	E	L	D	A	S	H	E	T	N	F	K	D	R	S	K	C	F	G	P	E	K	N	V	K	D	T	R	I	R	S	P
■ 1/chicken/EGY/H812/23	Egypt	K	L	Y	E	G	K	Q	F	P	I	E	L	D	A	S	H	E	T	N	F	K	D	R	T	K	C	S	G	P	E	K	N	V	K	D	T	R	I	Q	S	P
IBDV-CV98-2023	Egypt	K	L	Y	E	G	K	Q	F	S	I	E	L	D	A	S	H	E	T	N	F	K	D	R	T	K	C	S	-	-	-	-	-	-	-	-	-	-	-	-	-	-
IBDV-EGY-CV75-2023	Egypt	K	L	Y	E	G	K	Q	F	P	I	E	L	D	A	S	H	E	T	N	F	K	E	R	T	K	C	S	-	-	-	-	-	-	-	-	-	-	-	-	-	-
IBDV-EGY-F647-2023	Egypt	K	L	Y	K	R	N	Q	I	P	I	E	L	D	A	S	H	E	T	N	F	K	D	R	T	K	C	S	-	-	-	-	-	-	-	-	-	-	-	-	-	-
IBDV-EGY-F649-9-2023	Egypt	K	L	Y	E	G	K	Q	F	P	I	E	L	D	A	S	H	E	T	N	F	K	D	R	T	K	C	S	-	-	-	-	-	-	-	-	-	-	-	-	-	-
IBDV-F658-2023	Egypt	K	L	Y	E	G	K	Q	F	P	I	E	L	D	A	S	H	E	T	N	F	K	D	S	T	K	C	S	-	-	-	-	-	-	-	-	-	-	-	-	-	-
LN-2020-2	China	K	L	Y	E	G	K	Q	F	P	I	E	L	D	T	S	H	E	T	N	F	K	D	R	T	K	C	S	G	P	E	K	N	V	K	D	T	R	I	Q	N	Q
SHG352	China	K	L	H	E	G	K	Q	F	P	I	E	L	D	A	S	H	E	T	N	F	R	D	R	T	K	C	S	G	P	E	K	N	V	K	D	T	R	I	Q	S	Q
FJ2021	China	K	L	Y	E	G	K	Q	F	P	I	E	L	D	A	S	H	E	T	N	F	K	D	R	T	K	C	S	G	P	D	K	N	V	K	D	T	R	I	Q	S	Q
HB202201	China	K	L	Y	E	G	K	Q	F	P	I	E	L	D	A	G	R	E	T	N	L	K	D	R	T	K	C	S	G	P	E	K	N	V	K	D	T	R	T	Q	S	Q
IBD/SD/LY/CN/01/2020	China	K	L	Y	E	G	K	Q	F	P	I	E	L	D	A	S	H	E	T	N	F	R	D	R	T	K	C	S	G	P	E	K	N	V	K	D	T	R	T	Q	S	Q
QZ191002	China	K	L	Y	E	G	K	H	F	P	I	E	L	D	A	S	H	E	T	D	F	R	D	R	T	K	S	S	G	S	E	R	N	V	K	D	T	R	T	Q	S	Q
SHG358	China	K	L	Y	E	G	K	Q	F	P	I	V	L	D	A	S	H	E	A	N	F	R	D	R	T	K	C	S	G	S	E	K	N	V	K	D	T	K	T	Q	S	Q
ZD-2018-1	China	K	L	Y	E	G	K	Q	F	P	I	E	I	H	A	S	H	E	T	N	F	R	D	R	T	K	C	S	G	S	E	K	N	V	R	D	T	R	T	Q	S	Q
UPM1432/2019	Malaysia	R	L	Y	E	G	K	Q	F	P	I	E	L	D	A	S	H	E	T	N	F	R	D	R	T	K	S	S	G	S	E	K	N	V	K	D	T	R	I	Q	S	Q
IBD16Hen01	China	K	L	Y	E	G	K	Q	F	P	I	E	L	D	A	S	H	E	T	N	F	R	D	R	T	K	C	S	G	S	E	K	N	V	K	D	T	R	T	Q	S	Q
SHG19	China	K	L	Y	E	G	K	Q	F	P	I	E	L	D	A	S	H	E	T	N	F	R	D	R	T	K	C	S	G	S	E	K	N	V	K	D	T	R	T	Q	S	Q
HB-2020-1	China	K	L	Y	E	G	K	Q	F	P	I	E	L	D	A	S	H	E	T	N	F	R	D	R	T	K	C	S	G	S	E	K	Y	V	K	D	T	R	T	Q	S	Q
GD-2	China	K	L	Y	E	G	K	Q	F	P	I	E	L	D	A	S	H	E	T	N	F	R	D	R	T	K	C	S	G	S	E	K	N	V	K	D	T	R	T	Q	S	Q
Hb06v	China	K	F	Y	E	G	K	Q	F	P	I	E	L	D	A	S	H	E	T	N	F	R	D	R	T	K	C	S	G	S	E	K	N	I	K	D	A	R	T	Q	S	Q
GD2021-17	China	K	L	Y	E	G	K	Q	F	P	I	E	L	D	A	S	H	E	T	N	F	R	D	R	T	K	C	S	G	S	E	K	N	V	K	N	T	R	T	Q	S	Q
LN-2020-1	China	K	L	Y	E	G	K	Q	F	P	I	E	L	D	A	S	H	E	T	N	F	R	D	R	T	K	C	S	G	S	E	K	N	V	K	D	T	R	T	Q	S	Q
FJ2019	China	K	L	Y	E	G	K	Q	F	P	V	E	L	D	A	S	H	E	T	N	F	R	D	R	T	R	C	S	G	S	E	K	N	V	K	D	T	R	T	Q	S	Q
